# Strain, Doping, and Electronic Transport of Large Area Monolayer MoS_2_ Exfoliated on Gold and Transferred to an Insulating Substrate

**DOI:** 10.1021/acsami.1c05185

**Published:** 2021-06-24

**Authors:** Salvatore Ethan Panasci, Emanuela Schilirò, Giuseppe Greco, Marco Cannas, Franco M. Gelardi, Simonpietro Agnello, Fabrizio Roccaforte, Filippo Giannazzo

**Affiliations:** † CNR-IMM, Strada VIII, 5 95121, Catania, Italy; ‡ Department of Physics and Astronomy, University of Catania, Via Santa Sofia 64, 95123 Catania, Italy; § Department of Physics and Chemistry Emilio Segrè, University of Palermo, Via Archirafi 36, 90123 Palermo, Italy; ∥ ATeN Center, Università degli Studi di Palermo, Viale delle Scienze, Edificio 18, 90128 Palermo, Italy

**Keywords:** MoS_2_, gold-assisted exfoliation, Raman, photoluminescence, conductive atomic force microscopy, doping, strain

## Abstract

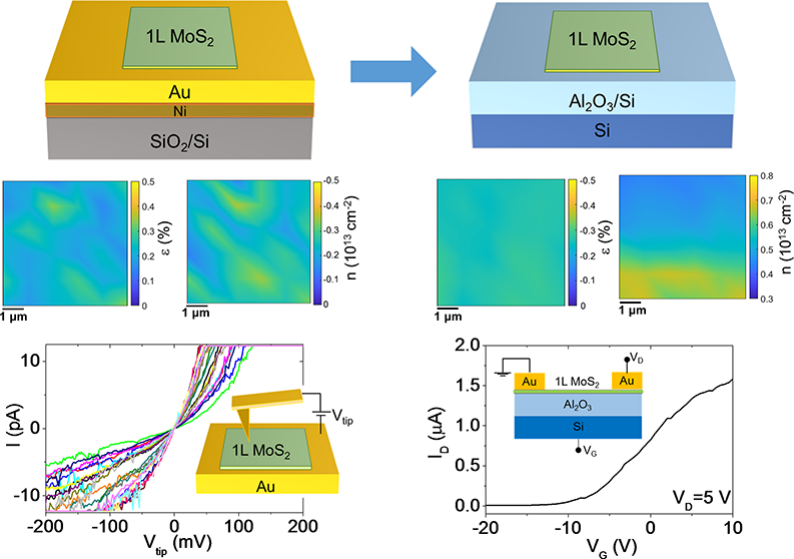

Gold-assisted mechanical exfoliation currently represents a promising method to separate ultralarge (centimeter scale) transition metal dichalcogenide (TMD) monolayers (1L) with excellent electronic and optical properties from the parent van der Waals (vdW) crystals. The strong interaction between Au and chalcogen atoms is key to achieving this nearly perfect 1L exfoliation yield. On the other hand, it may significantly affect the doping and strain of 1L TMDs in contact with Au. In this paper, we systematically investigated the morphology, strain, doping, and electrical properties of large area 1L MoS_2_ exfoliated on ultraflat Au films (0.16–0.21 nm roughness) and finally transferred to an insulating Al_2_O_3_ substrate. Raman mapping and correlative analysis of the E′ and A_1_′ peak positions revealed a moderate tensile strain (ε ≈ 0.2%) and p-type doping (*n* ≈ −0.25 × 10^13^ cm^–2^) of 1L MoS_2_ in contact with Au. Nanoscale resolution current mapping and current–voltage (*I*–*V*) measurements by conductive atomic force microscopy (C-AFM) showed direct tunneling across the 1L MoS_2_ on Au, with a broad distribution of tunneling barrier values (Φ_B_ from 0.7 to 1.7 eV) consistent with p-type doping of MoS_2_. After the final transfer of 1L MoS_2_ on Al_2_O_3_/Si, the strain was converted to compressive strain (ε ≈ −0.25%). Furthermore, an n-type doping (*n* ≈ 0.5 × 10^13^ cm^–2^) was deduced by Raman mapping and confirmed by electrical measurements of an Al_2_O_3_/Si back-gated 1L MoS_2_ transistor. These results provide a deeper understanding of the Au-assisted exfoliation mechanism and can contribute to its widespread application for the realization of novel devices and artificial vdW heterostructures.

## 1 Introduction

Semiconducting transition metal dichalcogenides (TMDs) are a class of two-dimensional (2D) layered materials with the general chemical formula MX_2_, where M is a transition metal (Mo, W, ...) and X is a chalcogen (S, Se, ...), which are characterized by strong (covalent) in-plane bonds and weak van der Waals (vdW) interactions between the layers.^1^ In particular, due to its abundance in nature and good stability under ambient conditions, molybdenum disulfide (MoS_2_) has been the most widely investigated TMD for potential applications in electronics, optoelectronics, photodetection, and sensing.^2−5^ In its bulk form, MoS_2_ shows an indirect band gap of 1.2 eV, whereas the monolayer counterpart exhibits a direct band gap of ∼1.8 eV.^6−10^ The sizable band gap, combined with a low dielectric constant, has made MoS_2_ a potential candidate to replace silicon as a channel material in ultrathin body field effect transistors for next-generation CMOS applications.^11−13^ Furthermore, the band-gap tunability of MoS_2_, obtained by tailoring the number of layers,^14^ strain,^15^ or dielectric environment,^16^ offers many possibilities to realize new concept beyond-CMOS electronic devices.^17^

Many of the MoS_2_ device prototypes demonstrated so far have been fabricated using monolayer flakes or few-layer flakes obtained by mechanical exfoliation from bulk molybdenite. In spite of the reported progress in the scalable exfoliation (lithiation/sonication,^18^ electrochemical exfoliation,^19^ etc.) and large area deposition of TMDs (chemical vapor deposition,^20,21^ molecular beam epitaxy,^22^ pulsed laser deposition,^23^ etc.), mechanical exfoliation still remains a method of choice for investigating basic physical phenomena and demonstrating new device concepts, due to the superior quality of the material produced by this approach.^24,25^

To overcome the limitations represented by the small (micrometer) size of the exfoliated flakes and the lack of reproducibility in the thickness, appropriate strategies allowing increase of the exfoliated monolayer area have been recently elaborated. In particular, the so-called “gold-assisted” mechanical exfoliation approach showed the possibility of separating large area (cm^2^) monolayer MoS_2_ (1L MoS_2_) from a bulk crystal stamp by exploiting the strong affinity between a gold film and the topmost sulfur atoms of MoS_2_.^26−29^ The exfoliation process from the bulk stamp can be repeated many times, producing flakes with reproducible geometry at each exfoliation step, with a size limited only by the dimensions of currently available bulk samples. The Au/1L MoS_2_ stack can be also transferred to insulating substrates, and after Au removal by chemical etching, the large area MoS_2_ film exhibits electronic properties fully comparable with those of the semiconducting MoS_2_ flakes obtained by conventional Scotch tape exfoliation.^27,30^ The gold-assisted exfoliation approach has been shown to be effective also with other common TMDs (such as MoSe_2_, WS_2_, WSe_2_, MoTe_2_, WTe_2_, and GaSe)^26,28,31,32^ as well as with other 2D crystals, including metal monochalcogenides (e.g., GaS), black phosphorus, black arsenic, metal trichlorides (e.g., RuCl_3_), and magnetic compounds (e.g., Fe_3_GeTe_2_).^32^ Furthermore, it has been recently proposed as a general approach to produce large area heterostructures of different TMDs with outstanding electronic quality by sequentially stacking the exfoliated monolayers.^31^ The 1L MoS_2_/Au system is also currently the object of increasing interest for technological applications. As an example, the Au/1L MoS_2_/Au heterojunctions hold great promise for nonvolatile switching memory applications.^33,34^ After the first “atomristor” demonstration using a CVD grown monolayer MoS_2_ sandwiched between two inert Au contacts,^33^ the possibility to implement such a system using high quality and large area 1L MoS_2_ mechanically exfoliated on Au has been also recently explored.^34^

In the past few years, several morphological and spectroscopic investigations have been reported on the Au/MoS_2_ system, with the aim of deeply understanding the mechanisms of the Au-assisted exfoliation and to maximize the monolayer fraction and the lateral size of the obtained MoS_2_ films. In particular, the 1L exfoliation yield was shown to be strongly influenced by the gold surface morphology and its exposure to the air before exfoliation.^26^ Due to the strong vdW interaction at the MoS_2_/Au interface, the Au morphology may significantly affect also the doping and strain in 1L MoS_2_, as shown by Raman analyses.^29^ An increase of the density of states (DOS) at the Fermi energy (i.e., a metallic character) was predicted by ab initio simulations of the MoS_2_/Au heterostructure as compared to semiconducting freestanding MoS_2_.^26^ Such an increased DOS in 1L MoS_2_ associated with the underlying Au was also demonstrated by electrochemical characterization of the MoS_2_/Au system.^26^ Recent investigations have been also reported on the evolution of the electronic properties of 1L MoS_2_ and other TMDs (MoSe_2_, MoTe_2_) exfoliated on Au surfaces during thermal annealing processes at temperatures up to 500 °C.^35,36^ These studies revealed the occurrence of a phase transition from the 2H semiconductor to the 1T conductive phase of MoS_2_ after the annealing, as a result of the interplay of charge transfer and strain induced by the Au substrate and defects in the MoS_2_ layer.

In this context, a systematic study on the evolution of the structural and electronic properties of 1L MoS_2_ in the different stages of the Au-assisted exfoliation process, i.e., after adhesion with gold and after final transfer to an insulating substrate, is still missing in the literature, and it would be highly desirable, considering the interest in this material system. In our work, we investigated the morphology, strain, doping, and electrical properties of 1L MoS_2_ exfoliated on ultraflat Au films and finally transferred to an Al_2_O_3_/Si substrate. To this purpose, micro-Raman and microphotoluminescence mapping experiments have been carefully executed and a method for the evaluation of doping and strain status has been applied in an original way. The correlative analysis of the E′ and A_1_′ Raman peak positions in spatial mapping revealed a moderate tensile strain (∼0.2%) and p-type doping (0.25 × 10^13^ cm^–2^) of 1L MoS_2_ in contact with Au. Nanoscale resolution current mapping and current–voltage (*I*–*V*) measurements by conductive atomic force microscopy (C-AFM) showed direct tunneling across the 1L MoS_2_ on Au, with a broad distribution of tunneling barrier values (Φ_B_ from 0.7 to 1.7 eV) indicating wide point-to-point variations of MoS_2_ p-type doping. After the final transfer of 1L MoS_2_ on Al_2_O_3_/Si and complete removal of the Au film, the strain was converted to compressive (−0.25%) and an n-type doping of ∼0.5 × 10^13^ cm^–2^ was observed by Raman spectroscopy and confirmed by electrical measurements on an Al_2_O_3_/Si back-gated 1L MoS_2_ transistor.

## 2 Results and Discussion

The lateral extension and thickness uniformity of MoS_2_ monolayers exfoliated on a smooth gold surface were initially assessed. To this aim, a 15 nm thick Au film was deposited onto a SiO_2_/Si substrate by DC magnetron sputtering (as schematically reported in Figure S1 of the Supporting Information). Prior to Au deposition, a 10 nm thick Ni film was sputtered to improve the adhesion onto the SiO_2_. Beside ensuring an optimal adhesion to the SiO_2_ surface, the Ni interlayer was beneficial for achieving a very smooth surface of the Au overlayer, with a low root-mean-square (RMS) roughness of 0.16 nm, as deduced from the tapping mode atomic force microscopy (AFM) image reported in Figure S2 of the Supporting Information. Mechanical exfoliation of MoS_2_ was carried out on the fresh Au surface, i.e., immediately after the deposition, in order to avoid its contamination with adventitious carbon, which is known to reduce the interaction strength between S atoms and Au.^26^ By this procedure, very large area MoS_2_ films, mostly composed of a monolayer, were separated from the bulk crystal.

**Figure 1:**
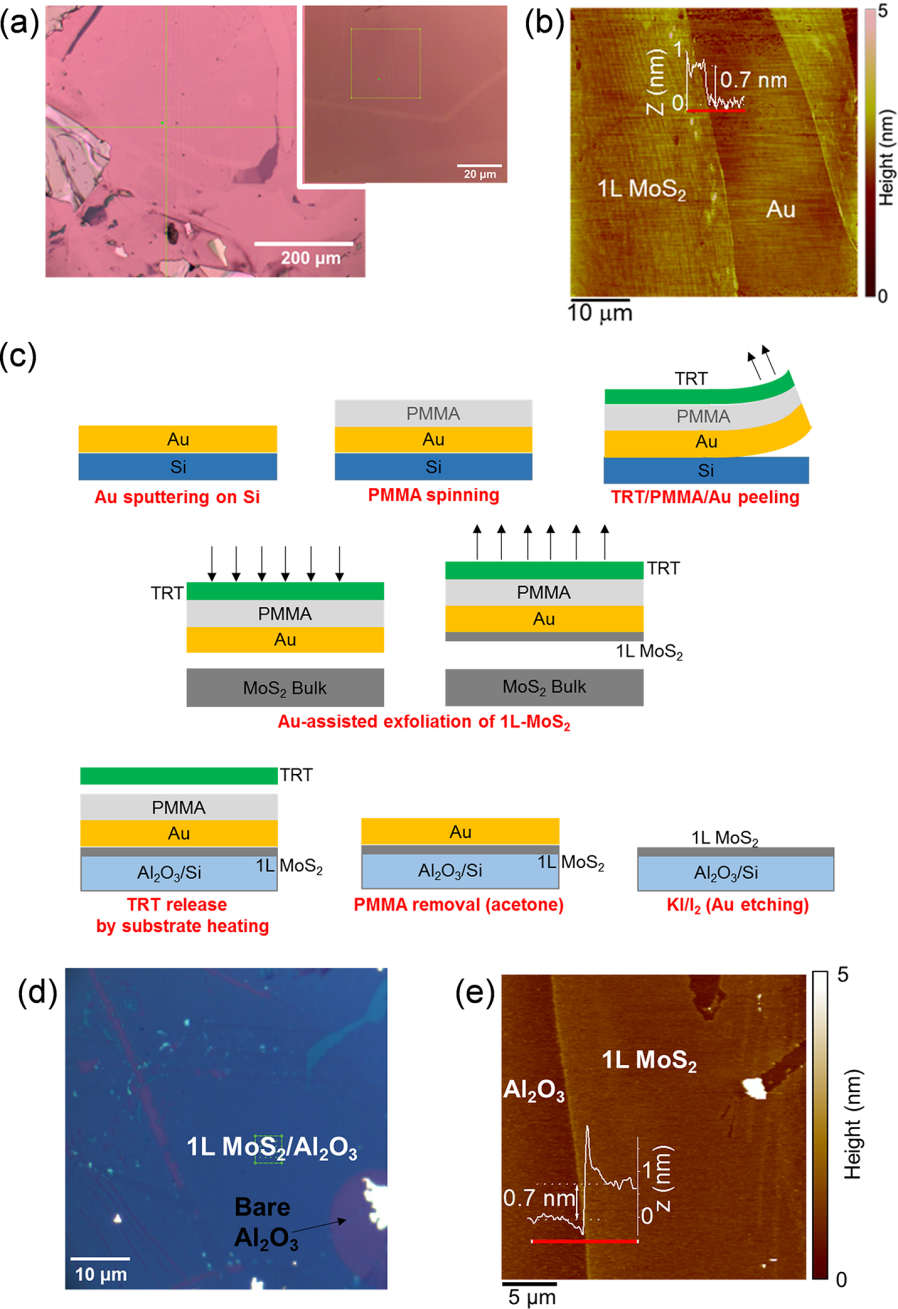
(a) Optical images at two different magnifications of exfoliated MoS_2_ on Au/Ni/SiO_2_. (b) AFM image of ultrathin MoS_2_ film with a fracture. The ∼0.7 nm step height in the height profile along the red line demonstrates the 1L thickness of MoS_2_ on Au. (c) Schematic illustration of the three steps for Au-assisted exfoliation of 1L MoS_2_ and transfer to Al_2_O_3_/Si substrate. (d) Optical image and (e) AFM morphology of transferred 1L MoS_2_ membrane on Al_2_O_3_/Si substrate and height line scan along the red line.

Figure 1a shows two optical images at different magnifications (10× and 100×, respectively) of the exfoliated MoS_2_ on the Au surface. The presence of an ultrathin MoS_2_ film extending for several hundred micrometers can be deduced from the color contrast in the lower magnification image, which also shows the presence of thicker MoS_2_ areas of smaller size and fractures of the MoS_2_ membrane (i.e., bare Au regions) due to the exfoliation process. The optical contrast difference between the uniform ultrathin MoS_2_ membrane and one of these fractures can be better visualized in the higher magnification image in the inset of Figure 1a. Furthermore, a typical tapping mode AFM image of a fracture of the MoS_2_ film is reported in Figure 1b. The ∼0.7 nm step height measured by the line profile in the inset is a direct confirmation of the 1L thickness of the MoS_2_ membrane. Furthermore, a higher resolution AFM image of 1L MoS_2_ partially covering the Au surface is reported in Figure S3 of the Supporting Information. The histogram of the height distribution extracted from this image shows very similar RMS values for the MoS_2_/Au (∼0.25 nm) and bare Au areas (∼0.28 nm), indicating a very conformal coverage by the MoS_2_ membrane.

After assessing the thickness uniformity of 1L MoS_2_ films exfoliated on gold, we investigated the transfer of these films to an insulating substrate, which is a mandatory requirement for most electronic applications. More specifically, a Si substrate covered by a 100 nm Al_2_O_3_ film was employed in this experiment, although the transfer procedure can be easily extended to other semiconductors or dielectric materials. Following the approach recently demonstrated by Liu et al.,^31^ the transfer procedure consisted of three different steps, schematically illustrated in Figure 1c. The first step was the fabrication of an ultraflat “gold tape”, consisting of a gold film on a polymer substrate. To this aim, a ∼100 nm thick Au layer was deposited by DC magnetron sputtering on an accurately precleaned silicon sample. Afterward, the Au surface was spin-coated by a protective PMMA layer and attached to a thermal release tape (TRT). By exploitation of the poor adhesion between Au and Si, the TRT/PMMA/Au stack was easily peeled from the silicon surface, and thus the desired “gold tape” was obtained. The surface of Au films prepared by this method is typically very flat,^37,38^ and it has already been demonstrated to be suitable for the exfoliation of large area monolayers of MoS_2_ and other TMDs.^31^ In particular a RMS roughness of 0.21 nm was evaluated with AFM on the peeled Au films on PMMA in our experiments (see Figure S4 of the Supporting Information), which is comparable with that of the Au/Ni film on SiO_2_. The TRT/PMMA/Au stamp with a fresh Au surface, i.e., immediately after peeling from Si, was used to exfoliate 1L MoS_2_ from a MoS_2_ bulk sample. The final step of the process was the transfer of 1L MoS_2_ on the target Al_2_O_3_/Si surface. This was achieved by pressing the TRT/PMMA/Au/1L MoS_2_ stack onto the Al_2_O_3_/Si substrate while heating at 120 °C to promote the TRT release, followed by PMMA removal and final chemical etching of the Au film (with KI/I_2_ solution). Figure 1d reports a typical optical microscopic image of the transferred MoS_2_ membrane on the Al_2_O_3_ surface. As compared to the case of 1L MoS_2_ exfoliated on gold (Figure 1a), a much sharper color contrast can be observed between the regions coated by the extended 1L MoS_2_ membrane (blue) and bare Al_2_O_3_ regions (violet), due to the favorable optical interference with the 100 nm Al_2_O_3_/Si substrate. Furthermore, the small regions coated by few-layer or multilayer MoS_2_ can be easily identified by the azure or bright color, respectively. Hence, the optical image provides useful information on the thickness uniformity of the transferred MoS_2_ film on a large area. Furthermore, a morphological AFM image of a sample region partially covered by the 1L MoS_2_ membrane is shown in Figure 1e.

**Figure 2:**
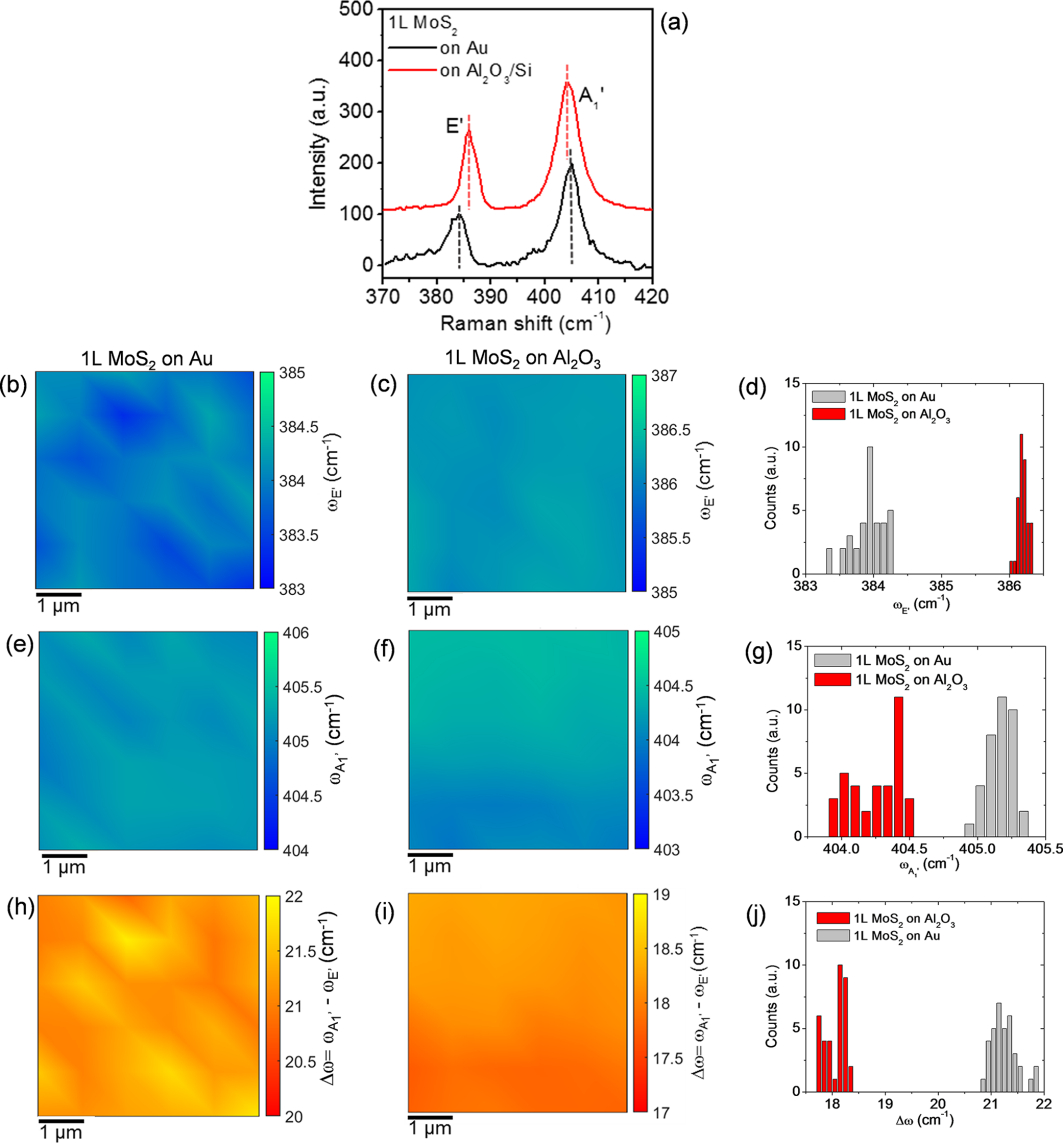
(a) Representative Raman spectra for 1L MoS_2_ on Au (black line) and on Al_2_O_3_/Si (red line). Color maps of E′ peak frequency values (ω_E′_) for 1L MoS_2_ on Au (b) and on Al_2_O_3_ (c) and corresponding histograms (d). Color maps of A_1_′ peak frequency values (ω_A_1_′_) for 1L MoS_2_ on Au (e) and on Al_2_O_3_ (f) and corresponding histograms (g). Color maps of peak frequency difference (Δω = ω_A_1_′_ – ω_E′_) for 1L MoS_2_ on Au (h) and on Al_2_O_3_ (i) and corresponding histograms (j).

The large area 1L MoS_2_ membranes exfoliated on Au and transferred onto Al_2_O_3_/Si were extensively investigated by micro-Raman mapping and photoluminescence (PL) spectroscopy, in order to evaluate the impact of the different substrates on relevant parameters, such as the doping and strain distributions. Figure 2a shows the comparison between two representative Raman spectra for 1L MoS_2_ on Au (black line) and on Al_2_O_3_/Si (red line), which indicated the characteristic E′ and A_1_′ peaks associated with the in-plane and out-of-plane MoS_2_ vibrational modes, respectively. It is worth noting that a peak frequency difference of Δω = 18 cm^–1^ is measured for our large area 1L MoS_2_ produced by Au-assisted exfoliation and transferred onto Al_2_O_3_, a value very similar to those reported for mechanically exfoliated or CVD-grown 1L MoS_2_ on common insulating substrates (such as SiO_2_).^25,39^ On the other hand, for the Au-supported 1L MoS_2_, the E′ peak exhibits a red shift and the A_1_′ peak exhibits a blue shift, resulting in a significantly larger value of Δω = 21 cm^–1^. It is well-known that E′ and A_1_′ spectral features are highly sensitive to strain and doping of 1L MoS_2_.^40,41^ In particular, a red shift of the E′ peak is typically observed with increasing tensile strain,^42,43^ followed by a peak splitting for large strain values.^29,44^ On the other hand, the A_1_′ peak is known to be sensitive to doping, and a blue (red) shift of its position is typically reported for p-type (n-type) doping of 1L MoS_2_.^45^ Hence, the increase in Δω for 1L MoS_2_ on Au/Ni/SiO_2_/Si can be ascribed to a change both in the strain and doping of the 2D membrane.

In order to extract relevant statistical information on the doping and strain uniformity of the 1L MoS_2_ membranes exfoliated on Au and transferred to the Al_2_O_3_/Si substrate, Raman mapping was carried out on both samples by collecting arrays of 6 × 6 spectra on a 5 × 5 μm^2^ area. Parts b and c of Figure 2 show the color maps of the E′ peak frequency (ω_E′_) in the scanned areas for 1L MoS_2_ on Au and Al_2_O_3_, respectively, while the comparison between the histograms of the ω_E′_ values in the two maps is shown in Figure 2d. Similarly, the color maps of the A_1_′ peak frequency (ω_A_1_′_) and corresponding histograms are reported in Figure 2e–g. Besides the individual peak positions, also their difference Δω = ω_A_1_′_ – ω_E′_ was calculated for all the collected Raman spectra. The color maps of the Δω values for 1L MoS_2_ on Au and Al_2_O_3_ are shown in Figure 2h,i, and the histograms of the Δω values are reported in Figure 2j.

The comparison between the color maps allows visualization of the spatial distribution of the ω_E′_, ω_A_1_′_, and Δω spectral features in the two different samples. As an example, it can be clearly deduced that the maxima of Δω for the Au-supported 1L MoS_2_ sample (Figure 2h) are correlated to the minima of the ω_E′_ map (Figure 2b), where the E′ peak is more red shifted. On the other hand, for the 1L MoS_2_ on Al_2_O_3_, the Δω map exhibits an almost uniform contrast, and the spatial variations are clearly correlated with those of the A_1_′ peak. The histograms in Figure 2d,g confirm on a large set of data the red shift of the E′ peak and the blue shift of the A_1_′ peak for 1L MoS_2_ on Au with respect to 1L MoS_2_ on Al_2_O_3_. It is also interesting to observe a significantly narrower spread of E′ values for the 1L MoS_2_ transferred to Al_2_O_3_, which can be ascribed to a more uniform strain distribution. By Gaussian fitting of the histograms, the average values and standard deviations of the peak frequencies and their difference have been obtained and are reported in Table 1.

**Table 1:** Average Values and Standard Deviations of E′ and A_1_′ Peak Frequencies (ω_E′_ and ω_A_1_′_) and Their Difference (Δω) and of Evaluated Strain and Doping for 1L MoS_2_ on Au and on Al_2_O_3_

	ω_E′_ (cm^–1^)	ω_A_1_′_ (cm^–1^)	Δω (cm^–1^)	ε (%)	*n* (10^13^ cm^–2^)
1L MoS_2_ on Au	383.9 ± 0.3	405.1 ± 0.1	21.2 ± 0.3	0.21 ± 0.06	–0.25 ± 0.06
1L MoS_2_ on Al_2_O_3_	386.2 ± 0.1	404.2 ± 0.1	18.1 ± 0.2	–0.25 ± 0.01	0.5 ± 0.09

In the following, the spatial distributions of strain ε (%) and doping *n* (cm^–2^) for 1L MoS_2_ on Au and on Al_2_O_3_ will be quantitatively evaluated from a correlative plot of the A_1_′ vs E′ peak frequencies for all Raman spectra in the maps of Figure 2. A similar approach, based on the correlative plot of the characteristic 2D and G peaks, has been widely employed for strain and doping quantification of monolayer graphene on different substrates.^46−50^ More recently such a method has been adopted by some authors also for 1L MoS_2_.^40,41,51^

**Figure 3:**
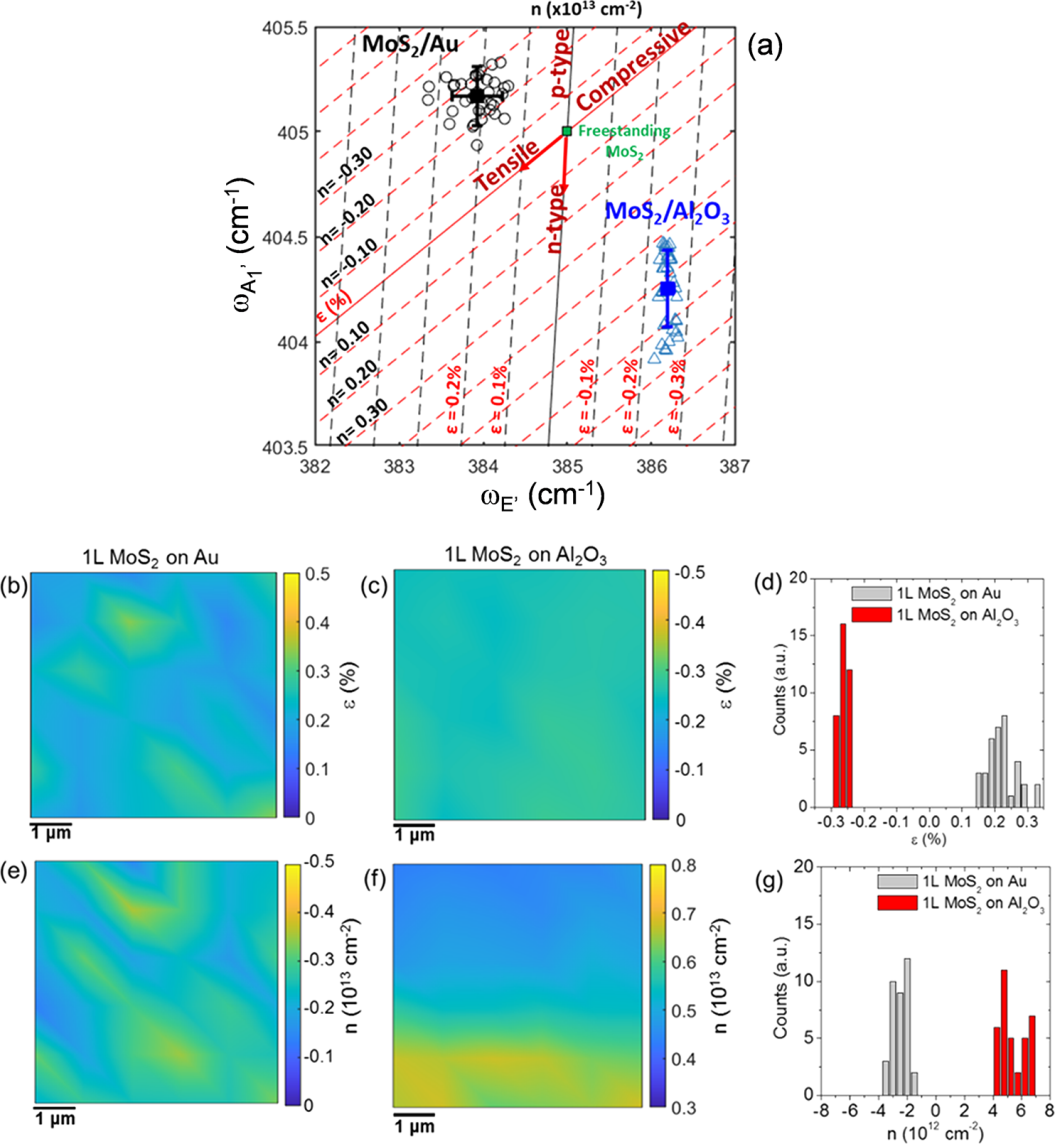
(a) Correlative plot of A_1_′ and E′ peak frequencies to evaluate biaxial strain and charge doping distributions in 1L MoS_2_ on Au (black circles) and on Al_2_O_3_ (blue triangles). The red (black) lines represent the strain (doping) lines for ideally undoped (unstrained) 1L MoS_2_, while the green square indicates the ω_E′_^0^ = 385 cm^–1^ and ω_A_1_′_^0^ = 405 cm^–1^ frequencies for freestanding 1L MoS_2_, taken as zero reference. The dashed red (black) lines parallel to the strain (doping) lines serve as guides to quantify the doping and strain values, respectively. Color maps of strain for 1L MoS_2_ on Au (b) and 1L MoS_2_ on Al_2_O_3_ (c) samples and histograms of the strain values (d). Color maps of doping for 1L MoS_2_ on Au (e) and 1L MoS_2_ on Al_2_O_3_ (f) and histograms of the doping values (g).

In Figure 3a, the black open circles represent the A_1_′ vs E′ pairs for all the Raman spectra collected on 1L MoS_2_ on Au, while the blue open triangles represent the data pairs for 1L MoS_2_ on Al_2_O_3_. The red and black lines represent the theoretical relations between the frequencies of the two vibrational modes at a laser wavelength of 532 nm in the ideal cases of a purely strained (strain line) and of a purely doped (doping line) 1L MoS_2_.^43,45^ The strain and doping lines cross in a point, corresponding to the ω_E′_^0^ and ω_A_1_′_^0^ frequencies for ideally unstrained and undoped 1L MoS_2_. In the following, the literature values of the peak frequencies for a suspended MoS_2_ membrane (ω_E′_^0^ = 385 cm^–1^ and ω_A_1_′_^0^ = 405 cm^–1^)^43^ have been kept as the best approximation to these ideal values, as substrate effects are excluded in this case. Starting from this reference point, the directions of increasing tensile strain and n-type doping are also indicated by the arrows along the two lines.

The ε and *n* values for each experimental point in Figure 3a can be evaluated from the combination of the linear relationships between the biaxial strain/charge doping and Raman shifts of the vibrational modes:
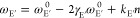
1a
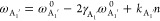
1b

Here, γ_E′_ = 0.68 and γ_A_1_′_ = 0.21 are the Grüneisen parameters for the two vibrational modes of 1L MoS_2_.^43,52,53^ The *k*_E′_ = −0.33 × 10^–13^ cm and *k*_A_1_′_ = −2.2 × 10^–13^ cm coefficients are the shift rates of Raman peaks as a function of the electron concentration *n* (in cm^–2^) in 1L MoS_2_, obtained by Raman characterization of electrochemically top-gated MoS_2_ transistors.^45^

In particular, the relation for the strain line can be obtained by solving the system of eqs 1a and 1b in the case of *n* = 0:
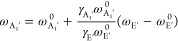
2whereas the doping line equation is obtained by the same procedure for ε = 0:
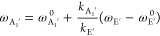
3

Hence,  and  are the slopes for the strain and doping lines, respectively. The dashed red lines parallel to the strain line (*n* = 0) and the dashed black lines parallel to the doping line (ε = 0) serve as guides to quantify the doping and strain values, respectively. They correspond to ±0.1% variations for the strain and ±0.1 × 10^13^ cm^–2^ variations for the doping. Since ω_E′_ is more sensitive to biaxial strain,^43^ the spacing between the dashed black lines parallel to the doping line is calculated from the E′ mode strain rate, 2γ_E′_ω_E′_^0^ = 5.2 cm^–1^/% . On the other hand, since the A_1_′ mode results are mainly influenced by charge doping,^43^ the spacing between the dashed red lines parallel to the strain line is calculated from the A_1_′ doping rate, *k*_A_1_′_.

The plot in Figure 3a shows that all the experimental data points for 1L MoS_2_ on Au are located above the strain line and in the left side with respect to the doping line. Hence, as compared to the reference case of a freestanding (suspended) 1L MoS_2_, our gold-supported 1L MoS_2_ films exhibit a tensile strain in the range from ∼0.1 to ∼0.3% and a p-type doping in the range from ∼0.1 × 10^13^ to ∼0.4 × 10^13^ cm^–2^. The average values of the strain (∼0.21%) and doping (∼0.25 × 10^13^ cm^–2^) are indicated by the black square in Figure 3a. A tensile biaxial strain, originating from the lattice mismatch between MoS_2_ and Au,^54,55^ has been recently observed in the case of 1L MoS_2_ exfoliated on Au also by other authors,^29^ who reported very large ε values up to 1.2%. The smaller tensile strain obtained in our samples is probably due to the very smooth surface of the gold films. The observed p-type doping of MoS_2_ in contact with Au is consistent with several recent reports of a p-type behavior induced by MoS_2_ functionalization with gold nanoparticles, adsorbates, or Au-based chemicals.^56−58^

On the other hand, the cloud of data for 1L MoS_2_ on Al_2_O_3_ is located in a region of the ε–*n* plane corresponding to a compressively strained and n-type doped film, with the strain values comprised in a narrow range around ∼ –0.25% and the electron density ranging from ∼0.4 × 10^13^ to ∼0.7 × 10^13^ cm^–2^. The compressive strain can be plausibly related to the transfer procedure and the adhesion properties of 1L-MoS_2_ with the Al_2_O_3_ surface. The observed n-type doping is consistent with the unintentional doping typically observed for MoS_2_ layers on insulating substrates and can be ascribed, in part, to charge transfer by adsorbed or interface trapped charges under ambient conditions, as well as to native defects of MoS_2_.

Through the solving of eqs 1a and 1b for all the data points of the ω_E′_ and ω_A_1_′_ maps, the corresponding color maps of the strain (Figure 3b,c) and doping (Figure 3e,f) for the two samples were obtained. The corresponding histograms of the strain and doping values are reported in parts d and g, respectively, of Figure 3. From the comparison of the strain and doping maps on 1L MoS_2_/Au, a correlation between the regions with higher tensile strain and those with higher p-type doping can be noticed. This suggests that both strain and p-type doping originate from a locally stronger interaction with Au. On the other hand, the compressive strain distribution appears very uniform in the 1L MoS_2_ membrane transferred onto Al_2_O_3_, without any clear correlation with the doping distribution. The average values and standard deviation of the strain and doping for the two different samples have been extracted by Gaussian fitting of the histograms in Figure 3d,g, and the obtained values have been reported in the Table 1. Obviously, the spatial resolution in these maps is limited by the laser spot size (∼1 μm). Furthermore, the concentration sensitivity (on the order of 10^12^ cm^–2^) is limited by the shift rate of the A_1_′ peak with doping concentration. Higher spatial resolution and sensitivity information on the doping distribution in the Au-supported 1L MoS_2_ will be deduced from conductive atomic force microscopy analyses reported later in this paper.

**Figure 4:**
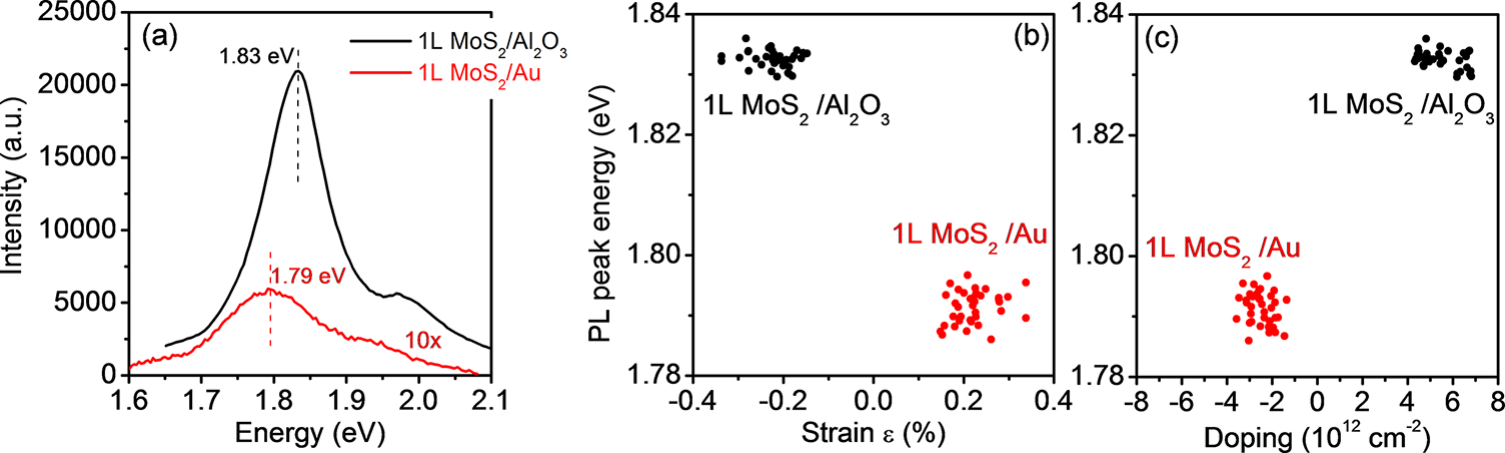
(a) Typical micro-PL spectra collected under excitation at 532 nm on 1L MoS_2_ on Au (with the intensity multiplied by a factor of 10) and 1L MoS_2_ transferred to Al_2_O_3_. Correlative plots of the PL peak energy with the strain (b) and doping values (c) deduced by PL and Raman mapping on the same sample area.

To further investigate the impact of the substrate/MoS_2_ interaction on the electronic properties of 1L MoS_2_, microphotoluminescence analyses were also performed using the 532 nm laser probe of the Raman equipment as the excitation source. Figure 4a shows the comparison between two representative PL spectra collected on two different samples under the same illumination conditions. It is worth noting that the large 1L MoS_2_ membrane produced by gold-assisted exfoliation and finally transferred onto Al_2_O_3_ exhibits a prominent peak at 1.83 eV, very similar to that observed for monolayer MoS_2_ obtained by the traditional mechanical exfoliation or deposited by CVD. On the other hand, a strongly reduced PL intensity is observed when the exfoliated 1L MoS_2_ membrane is still in contact with Au, together with a red shift of the main PL peak to 1.79 eV. The strong reduction of the PL intensity is consistent with the emission quenching reported by other authors for 1L MoS_2_ exfoliated on Au^26^ and for MoS_2_ functionalized with Au nanoparticles.^59^ This PL quenching can be explained in terms of a preferential transfer of photoexcited charges from MoS_2_ to Au. In addition, the tensile strain of the MoS_2_ layer in contact with Au can also play a role in the reduction of the PL yield.^43^ In order to obtain statistically relevant information, arrays of PL spectra have been collected on the two samples in the same areas previously probed by Raman mapping. The obtained color maps of the PL peak energy for 1L MoS_2_ on Au and after transfer on Al_2_O_3_ are reported in Figure S5 of the Supporting Information. Furthermore, Figure 4b,c shows the correlative plots of the PL peak energy with the strain and doping values deduced from Raman maps. These plots show a narrow distribution of the PL peak energies for both 1L MoS_2_/Au and 1L MoS_2_/Al_2_O_3_ samples. In particular, in Figure 4b the observed peak energy variations observed within each sample and the difference between the average values of the data points collected on the two different substrates are compatible with the PL peak shift rate as a function of the strain (∼100 meV/%) reported in the literature.^60^

**Figure 5:**
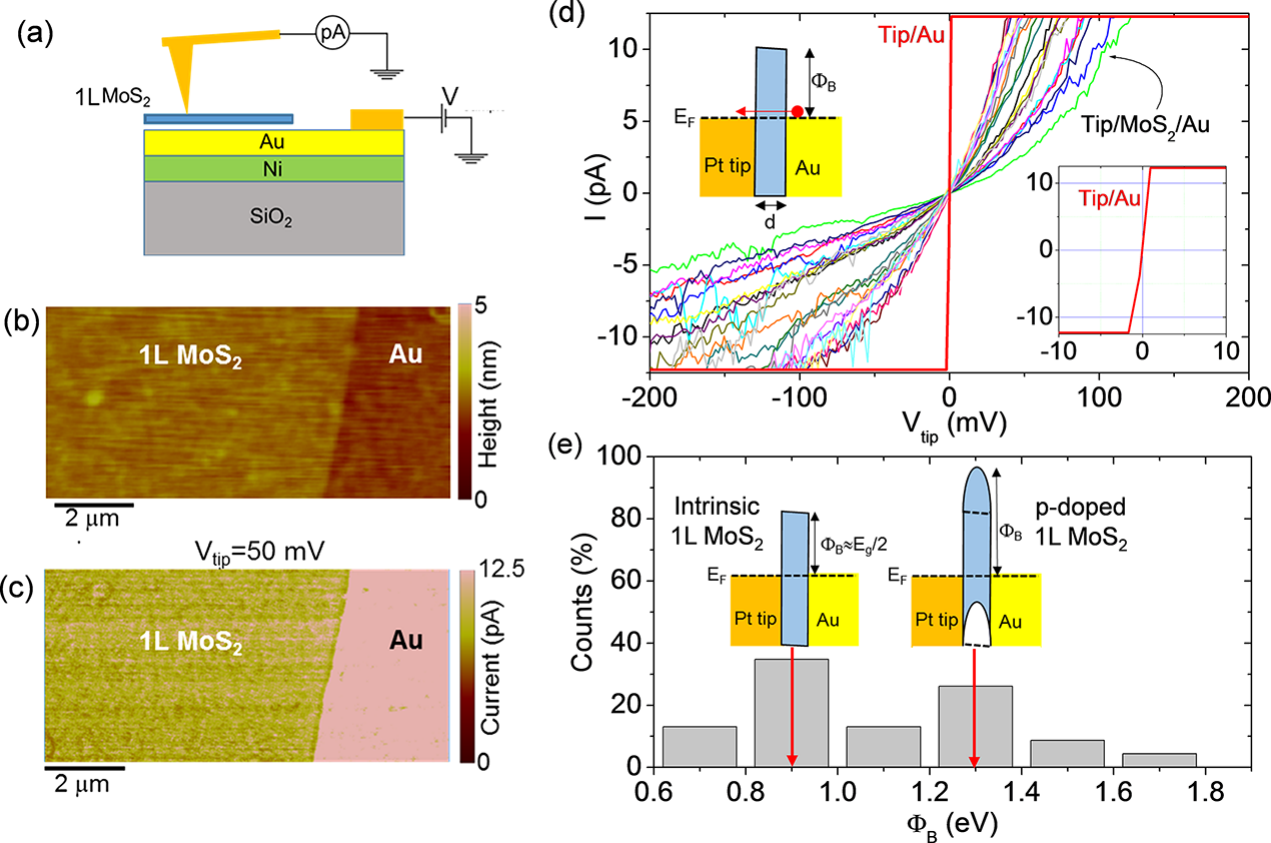
(a) Schematic illustration of C-AFM setup used for current mapping through 1L MoS_2_ film on Au. (b) Morphology of a sample region with the Au substrate partially covered by the 1L MoS_2_ film and (c) simultaneously measured current map on the same area (at *V*_tip_ = 50 mV). (d) Local *I*–*V*_tip_ curves measured with the Pt tip in contact with 1L MoS_2_ on Au and with the bare Au surface (red line). A detail of the *I*–*V*_tip_ curve measured on Au is reported in the right inset. A schematic band diagram for the tip/1L MoS_2_/Au metal/semiconductor/metal heterojunction is reported in the left inset. (e) Histogram of tunneling barrier values Φ_B_ evaluated from *I*–*V*_tip_ curves in (d), according to the direct tunneling mechanism. The band diagrams for intrinsic and p-type doped 1L MoS_2_ are schematically illustrated in the insets of (e).

The Raman mapping experiments reported so far provided information on the doping uniformity of 1L MoS_2_ based on the correlation between characteristic vibrational peaks and the carrier type and density. In the following, electrical measurements will be also employed to get further insight into the electronic transport in the 1L MoS_2_/Au system and after transfer to the insulating substrate. Recently, other groups used electrical scanning probe methods on TMDs transferred onto noble metal contacts to characterize the buried semiconductor/metal vdW interface.^61,62^ Here, we carried out a nanoscale resolution electrical characterization of the Au-supported 1L MoS_2_ membrane by C-AFM measurements^63^ to get further information on the doping uniformity in this ultrathin layer. To this aim, the current injection at the interface between the Au substrate and 1L MoS_2_ was probed at the nanoscale by a Pt coated Si tip, according to the configuration schematically illustrated in Figure 5a. The surface morphology in a sample region partially covered by 1L MoS_2_ is reported in Figure 5b, showing how the 1L MoS_2_ membrane conformally follows the smooth Au morphology. Furthermore, Figure 5c shows the simultaneously measured current map, collected by applying a DC bias *V*_tip_ = 50 mV between the Pt tip and the Au electrode (substrate). For this low bias value, the current level measured on the bare Au region reaches the current amplifier saturation limit, whereas appreciable lateral variations of the injected current through the 1L MoS_2_ membrane can be observed.

Such local variations of the injected current through the atomically thin membrane can be ascribed to the lateral inhomogeneities of MoS_2_ electronic properties. In this respect, it is worth noting that, since C-AFM analyses were performed in ambient conditions, an ultrathin water layer is adsorbed on the MoS_2_ surface and, consequently, the effective contact area is determined by the size of the water meniscus around the tip.^63^ As a matter of fact, the meniscus size is determined by the tip radius, the applied force, and the surface roughness. Hence, the smooth MoS_2_ surfaces of our samples and the application of a constant force during measurements result in a nearly constant effective contact area. To further investigate the current transport mechanisms, a set of local current–voltage (*I*–*V*_tip_) characteristics where acquired both on the bare Au surface and at different positions on the MoS_2_ film, as reported in Figure 5d. The *I*–*V*_tip_ curves measured by the Pt tip in contact with Au (see the red curve in Figure 5d) are very reproducible and exhibit an ohmic behavior with a very steep slope and current saturation at a few millivolts positive and negative biases (as shown in the right inset of Figure 5d). On the other hand, *I*–*V*_tip_ curves measured on MoS_2_ show significant variations at different positions. A possible reason for such variability can be the presence of nanoscale areas where 1L MoS_2_ is locally in contact with Au and areas where it is locally suspended between the Au grains, as recently reported by Velický et al.^29^ However, this scenario would imply a splitting of the A_1_′ peak in the Raman spectra, due to the very different substrate-related doping for the Au-supported and suspended 1L MoS_2_.^29^ Since such splitting is not observed in our Raman spectra, we excluded this effect in our 1L MoS_2_/Au samples.

In the following, the Pt tip in contact with 1L MoS_2_/Au is described as a metal/semiconductor/metal heterojunction, and the local *I*–*V*_tip_ curves in Figure 5d have been fitted with relevant current transport mechanisms across the ultrathin MoS_2_ barrier.

In Figure 5d all the curves measured on MoS_2_ show a linear behavior at low V_tip_ values, followed by a deviation from linearity at larger bias values. Furthermore, the slight asymmetry between positive and negative polarizations at larger biases can be ascribed to the different work functions of Pt and Au metals.

The linear behavior of the *I*–*V*_tip_ characteristics indicates direct tunneling (DT) as the most appropriate mechanism ruling current transport at low bias values.^64^ In particular, the tunneling current can be expressed as
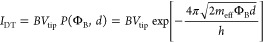
4where *B* is a prefactor (proportional to the tip contact area) and *P*(Φ_B_,*d*) is the direct tunneling probability, which is a function of the tunneling barrier thickness *d* (i.e., the MoS_2_ thickness) and its height Φ_B_, corresponding to the energy difference between the MoS_2_ conduction band and the Au Fermi level (see the schematic in the left inset of Figure 5d). Here *m*_eff_ = 0.35*m*_0_ is the electron effective mass in the transversal direction for 1L MoS_2_,^65^ and *h* is Planck’s constant. As a matter of fact, the thickness-dependent tunneling probability becomes unity when the MoS_2_ layer is absent (*d* = 0), i.e., when the tip is directly in contact with the Au substrate. Since current mapping and local *I*–*V* measurements have been performed using the same tip in a sample area including MoS_2_-covered and uncovered Au regions, the same value for the prefactor *B* were considered in the two cases. Hence, the experimental values of the local tunneling probability at different positions on MoS_2_ were estimated as the ratio between the slope of the *I*–*V* curves measured on MoS_2_ and the slope of the *I*–*V* characteristics measured on Au. Since the MoS_2_ layer is very conformal to the smooth Au morphology, we have assumed a laterally uniform 1L MoS_2_ barrier thickness of *d* = 0.65 nm (corresponding to the ideal value for 1L MoS_2_) over the C-AFM probed area. As a result, the local barrier height values have been extracted from the tunneling probabilities for each of the *I*–*V* curves in Figure 5d. The obtained histogram of the Φ_B_ values, reported in Figure 5e, shows a broad distribution, ranging from 0.70 ± 0.08 to 1.70 ± 0.08 eV, with two main components at ∼0.9 and ∼1.3 eV. In particular, the component at Φ_B_ ≈ 0.9 eV corresponds to a Fermi level located approximately at *E*_g_/2 with respect to the MoS_2_ conduction band, as schematically illustrated in the left inset of Figure 5e. It is worth noting that this value is very close to the ideal barrier height between Au and charge-neutral MoS_2_, given by Φ_B_ = *W* – χ, where *W* ≈ 5.1 eV is the gold work function and χ ≈ 4.2 eV is the electron affinity of 1L MoS_2_.^61^ This charge-neutral region can be ascribed to nanoscale areas where the p-type doping induced by the Au substrate is compensated by the presence of n-type doping impurities/adsorbates on the surface of MoS_2_.^66^ Due to the limited sensitivity of the Raman peak’s shift to doping concentration values of <10^12^ cm^–2^, such low doping areas could not be detected in Raman maps. On the other hand, the component at larger Φ_B_ values in the distribution of Figure 5e can be ascribed to higher p-type doping of 1L MoS_2_, which was detected also by Raman spectroscopy. Such local p-type doping due to the Au substrate induces an upward bending of the conduction and valence bands of MoS_2_ (schematically illustrated in the right inset of Figure 5e), which extends over a distance of the order of a few nanometers, defined by the Debye length in the 2D semiconductor.^67^

In addition to DT, trap-assisted-tunneling (TAT) is also expected to significantly contribute to the measured current by the C-AFM tip, due to the presence of a large density of native defects, such as sulfur vacancies, in exfoliated 1L MoS_2_ on Au.^68^ In particular, we have found that this transport mechanism is able to describe well the behavior of local *I*–*V*_tip_ curves in Figure 5d at higher bias values. Figure S6 of the Supporting Information shows the curves fitting with the TAT equation
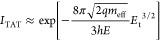
5where *E* is the electric field across 1L MoS_2_ and *E*_t_ is the trap energy below the conduction band edge. The resulting distribution of *E*_t_ values exhibits a peak at ∼0.8 eV, consistent with the results of other recent reports.^69^

**Figure 6:**
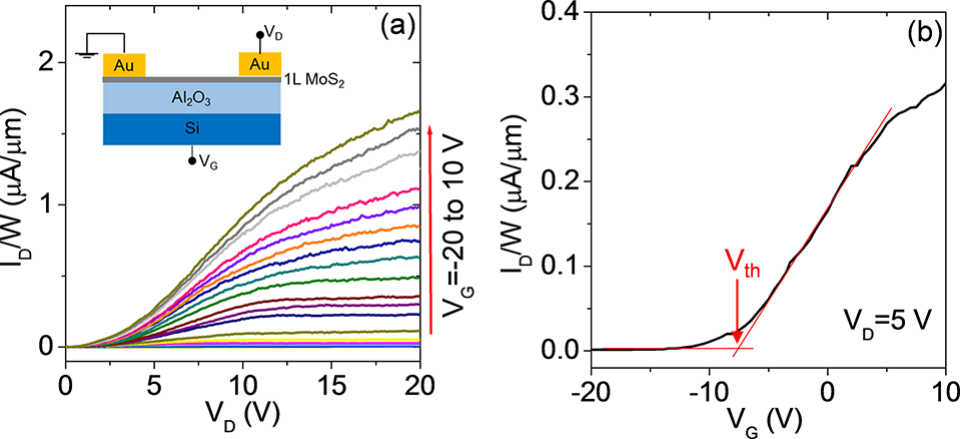
(a) Output and (b) transfer characteristics of a back-gated field effect transistor fabricated with Au-exfoliated 1L MoS_2_ transferred on Al_2_O_3_/Si. The device schematic is shown in the inset of (a).

Finally, the electronic transport in 1L MoS_2_ membrane transferred onto the Al_2_O_3_ dielectric surface has been investigated by electrical characterization of a field effect transistor (FET) with the Al_2_O_3_ (100 nm)/Si back gate and Au source and drain contacts (channel length *L* = 10 μm), as illustrated in the inset of Figure 6a. The output characteristics (drain current vs drain bias, *I*_D_–*V*_D_) of the device for different gate bias values ranging from *V*_G_ = −20 V to *V*_G_ = 10 V are shown in Figure 6a. At low drain bias (*V*_D_ < 3 V) current injection in the MoS_2_ channel is limited by the high Schottky barrier at Au/MoS_2_ contacts, whereas a linear behavior of the *I*_D_–*V*_D_ characteristics is observed at intermediate *V*_D_ values (from 3 to 10 V), followed by current saturation at higher voltages. The transfer characteristic (*I*_D_–*V*_G_) at a drain bias of *V*_D_ = 5 V (i.e., in the linear region of *I*_D_–*V*_D_ curves) is reported in Figure 6b (black line). The monotonic increase of *I*_D_ with *V*_G_ is the typically observed behavior for a transistor with an n-type MoS_2_ channel. A negative threshold voltage of *V*_th_ ≈ −8 V was evaluated by linear fitting of the *I*_D_–*V*_G_ curve and taking the intercept with the voltage axis, as indicated by the arrow in Figure 6b. Since *V*_th_ represents the bias necessary to deplete the n-type MoS_2_ channel, the electron density can be estimated as *n* = *C*_ox_|*V*_th_|/*q*, where *C*_ox_ = ε_0_ε_ox_/*t* is the Al_2_O_3_ capacitance per unit area, with ε_0_ the vacuum permittivity and ε_ox_ = 8 is the relative dielectric constant of the Al_2_O_3_ dielectric. The obtained carrier density *n* ≈ 3.1 × 10^12^ cm^–2^ is in reasonably good agreement with the carrier density values obtained by Raman mapping. Furthermore, the channel electron mobility (μ = 2.3 ± 0.1 cm^2^ V^–1^ s^–1^) has been properly evaluated excluding the effect of the contact resistance, as illustrated in Figure S7 of the Supporting Information.

This value is similar to the ones reported for back-gated monolayer MoS_2_ transistors without high-*k* encapsulation,^13^ where the mobility is limited by Coulomb scattering due to charged impurities. In the present case, part of these impurities may originate from the KI/I_2_ etching of gold involved in the transfer procedure. In this respect, appropriate strategies should be further elaborated to detach the exfoliated 1L MoS_2_ from the Au substrate without the use of chemical etching. These may include electrolytic delamination by hydrogen bubbling, used in the past to separate CVD graphene from copper^70^ and more recently to separate CVD TMDs from Au foils.^71^ Furthermore, a number of potential applications (such as memristor devices) are currently emerging, where the large area 1L MoS_2_ on gold can be directly employed without any need for transfer. In these cases, the exfoliated MoS_2_ membrane retains its excellent crystal quality.

## 3 Conclusion

In summary, large area (cm^2^) 1L MoS_2_ membranes have been exfoliated on very flat gold films and transferred to an insulating Al_2_O_3_/Si substrate. For 1L MoS_2_ in contact with Au, Raman mapping revealed a spatially inhomogeneous distribution of tensile strain (in the range from ∼0.1 to ∼0.3%) and p-type doping (from ∼0.1 × 10^13^ to ∼0.4 × 10^13^ cm^–2^), with a correlation between regions showing higher strain and doping. The electrical properties of Au-supported MoS_2_ were probed at the nanoscale by C-AFM, showing direct tunneling across the ultrathin 1L MoS_2_, with a broad distribution of tunneling barrier values (Φ_B_ from 0.7 to 1.7 eV) consistent with an inhomogeneous p-type doping of MoS_2_. After the final transfer of 1L MoS_2_ on Al_2_O_3_/Si, the strain was converted to compressive (ε ≈ −0.25%) with a very uniform distribution. Furthermore, an n-type doping (*n* ≈ 0.5 × 10^13^ cm^–2^) was deduced by Raman mapping and confirmed by electrical measurements of an Al_2_O_3_/Si back-gated 1L MoS_2_ transistor. These results provide a deeper understanding of the properties of large area 1L MoS_2_ produced by Au-assisted exfoliation, and they will contribute to the widespread application of this outstanding quality material in the demonstration of novel device concepts and synthetic van der Waals heterostructures.

## 4 Materials and Methods

### 4.1 Sample Preparation

The deposition of Ni (10 nm)/Au (15 nm) on a SiO_2_ (900 nm)/Si sample was carried out by DC magnetron sputtering using Quorum equipment. The base vacuum in the chamber was ∼10^–5^ mbar, while during the deposition process the pressure was about 10^–4^–10^–3^ mbar. The same equipment was employed to deposit 100 nm of Au on a Si sample for the preparation of the gold tape with the peeling technique (see Figure 2). PMMA (200 K, 0.5 μm) was spin-coated on Au and tempered at 150 °C. A Nitto Denko thermal release tape (with 120 °C release temperature) was used for the handling of the PMMA/Au gold tape. The 100 nm Al_2_O_3_ insulator on Si (used as a final substrate for 1L MoS_2_ transfer) was deposited by DC-pulsed RF reactive sputtering.

### 4.2 AFM and C-AFM Analyses

Morphological analyses of the Au/Ni substrates and of the exfoliated MoS_2_ films were carried out by tapping mode atomic force microscopy (AFM) using DI3100 equipment by Bruker with Nanoscope V electronics. Sharp silicon tips with a curvature radius of 5 nm were used for these measurements. C-AFM measurements were carried out with the same AFM system equipped with the TUNA module and using Pt coated Si tips. All the AFM and C-AFM analyses were carried out at room temperature and under ambient atmosphere.

### 4.3 Micro-Raman Spectroscopy and Microphotoluminescence

Raman spectroscopy and PL measurements were carried out by using a Horiba HR-Evolution micro-Raman system with a confocal microscope (100× objective) and a laser excitation wavelength of 532 nm. The laser power used for these analyses was filtered with a neutral density filter at 1% ensuring no sample degradation. A grating of 1800 lines/mm was employed to acquire Raman spectra in a range from 150 to 650 cm^–1^, while a grating of 600 lines/mm was used to acquire photoluminescence spectra in a range from 10 to 5500 cm^–1^. All the spectra were calibrated with respect to the silicon peak at 520.7 cm^–1^.

### 4.4 Field Effect Transistor Preparation and Characterization

A back-gated 1L MoS_2_ field effect transistor was fabricated with the Au-exfoliated film transferred onto Al_2_O_3_ (100 nm)/Si by sputtering Au source/drain contacts with a shadow mask. The contact spacing, i.e., the channel length, was *L* = 10 μm. The output and transfer characteristics of the transistor were measured in dark conditions by using a Cascade Microtech probe station with an Agilent 4156b parameter analyzer.

## Supporting Information Available

Schematic illustration of procedure for 1L MoS_2_ exfoliation on Au/Ni/SiO_2_; AFM images and histograms of height distribution for Au/Ni/SiO_2_ substrate, peeled Au on PMMA, and 1L MoS_2_ partially covering the Au/Ni/SiO_2_ substrate; maps of PL peak energy from spectra collected on 1L MoS_2_ exfoliated on Au and after transfer on Al_2_O_3_; fit of local *I*−*V*_tip_ curves by the trap-assisted-tunneling (TAT) mechanism; evaluation of the contact resistance and field-effect mobility of the back-gated MoS_2_ field effect transistor (PDF)
