# Correction to “Strain, Doping, and Electronic Transport of Large Area Monolayer MoS_2_ Exfoliated on Gold and Transferred to an Insulating Substrate”

**DOI:** 10.1021/acsami.2c12608

**Published:** 2022-07-29

**Authors:** Salvatore Ethan Panasci, Emanuela Schilirò, Giuseppe Greco, Marco Cannas, Franco M. Gelardi, Simonpietro Agnello, Fabrizio Roccaforte, Filippo Giannazzo

In the original version of this article, the eqs 1a, 1b, and 4 contained typographical errors. The correct equations are as follows:
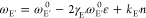
1a
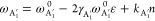
1b
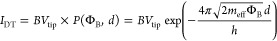
4

These errors are typographical and have no effect on discussion and conclusions, as the correct equations were used in the data analysis to obtain Figures 3 and 5.

