# Supplementary material for: Strain, Doping, and Electronic Transport of Large Area Monolayer MoS2 Exfoliated on Gold and Transferred to an Insulating Substrate
Source: ACS Appl Mater Interfaces. 2021 Jun 24;13(26):31248–59. doi: 10.1021/acsami.1c05185 (PMC9280715; doi:10.1021/acsami.1c05185)
Supplement: Supplementary file 1 — am1c05185_si_001.pdf [file am1c05185_si_001.pdf]

Supporting Information

Strain, doping and electronic transport of large area monolayer MoS_2 exfoliated on gold and transferred to an insulating substrate

Salvatore Ethan Panasci[1,2], Emanuela Schilirò[1], Giuseppe Greco[1], Marco Cannas[3], Franco M. Gelardi[3], Simonpietro Agnello[3,1,4], Fabrizio Roccaforte[1], Filippo Giannazzo[1*]

[1] *CNR-IMM, Strada VIII, 5 95121, Catania, Italy*

[2] *Department of Physics and Astronomy, University of Catania, Via Santa Sofia 64, 95123 Catania, Italy*

[3] *Department of Physics and Chemistry Emilio Segrè, University of Palermo, Via Archirafi 36, 90123 Palermo, Italy*

[4] *ATeN Center, Università degli Studi di Palermo, Viale delle Scienze, Edificio 18, 90128 Palermo, Italy*

E-mail: filippo.giannazzo@imm.cnr.it

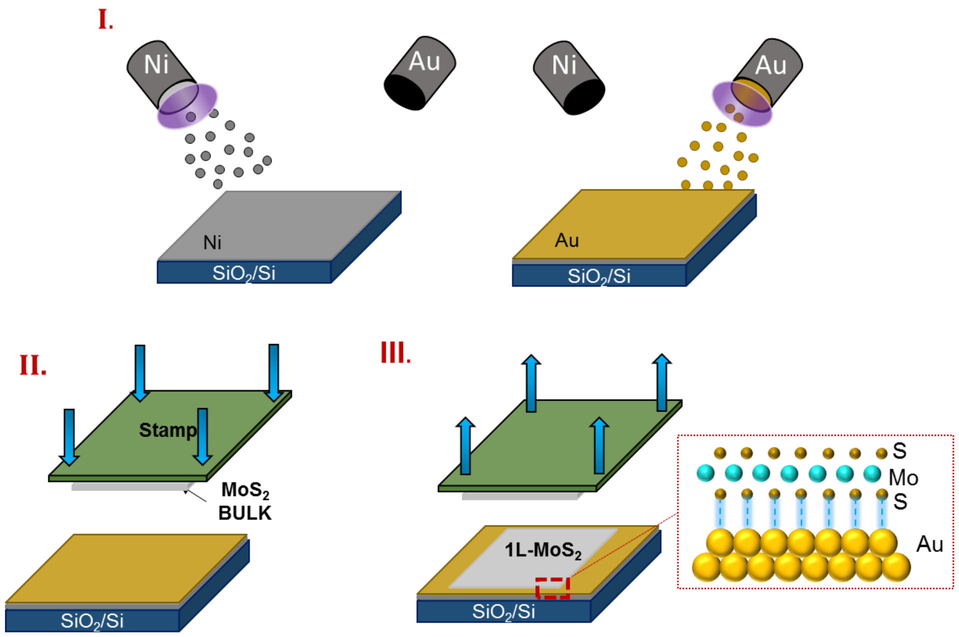

Figure S1 Schematic illustration of the procedure for monolayer (1L) MoS_2 exfoliation on Au/Ni/SiO_2. (I) Sequential sputtering of Ni (adhesion layer) and Au on the SiO_2/Si substrate. (II) Pressing of bulk MoS_2 on the Au surface. (III) Separation of 1L-MoS2 due to the strong interaction between S and topmost Au atoms.

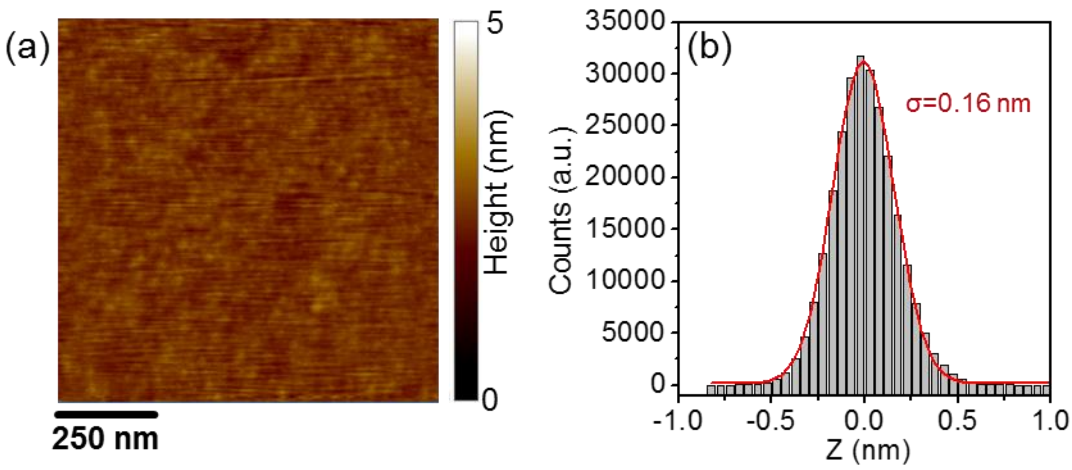

Figure S2 (a) Tapping mode Atomic Force Microscopy image of the Au surface morphology in Au/Ni/SiO_2 samples. (b) Z-values distribution and Gaussian fit, with the standard deviation (roughness) σ=0.16 nm.

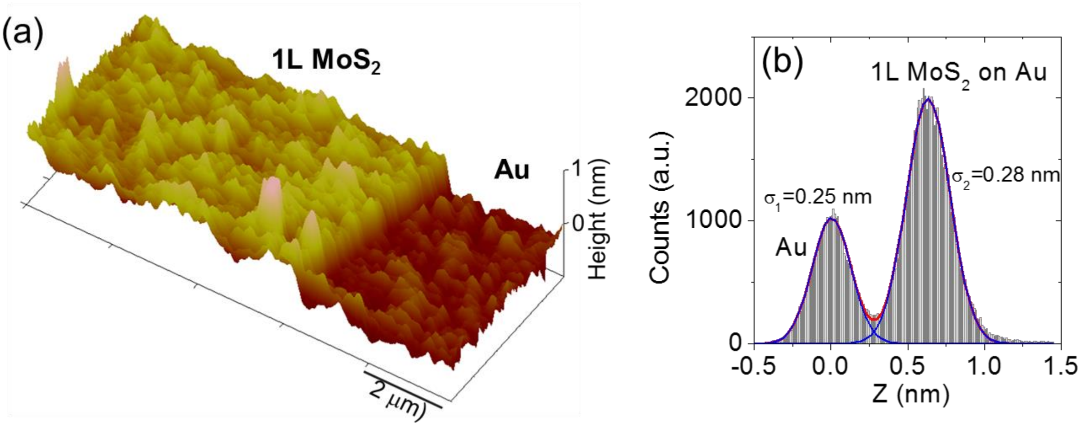

Figure S3 1L MoS_2 exfoliated on Au. (a) AFM morphology of a region where MoS_2 partially covers the Au surface. (b) Corresponding histogram of height distribution, showing two components, associated to the bare Au region and to 1L MoS_2/Au. Very small and comparable values of roughness (σ_1=0.25 nm and σ_2=0.28 nm) can be deduced for both areas.

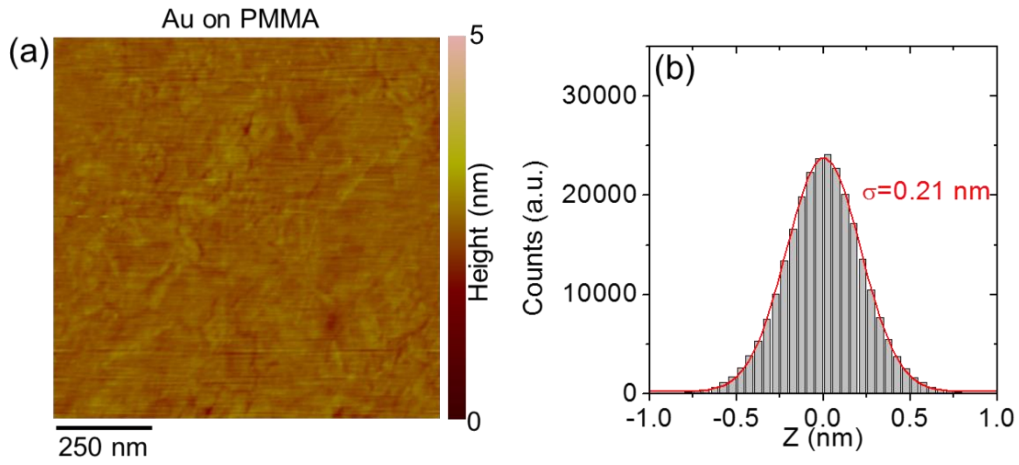

Figure S4 (a) AFM morphology of the peeled gold on PMMA and (b) corresponding histogram of height distribution, showing a very small roughness of 0.21 nm, comparable with that of the Au/Ni film on SiO_2.

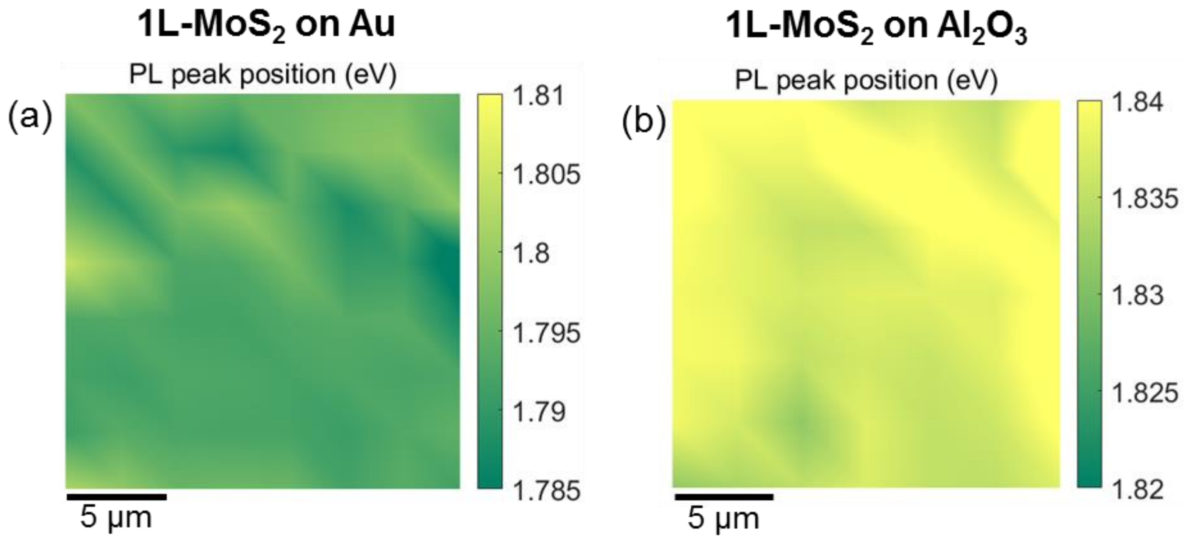

Figure S5 Colour maps of the PL peak energy obtained from arrays of microPL spectra collected on 1L MoS_2 exfoliated on Au (a) and after transfer on Al_2O_3 (b).

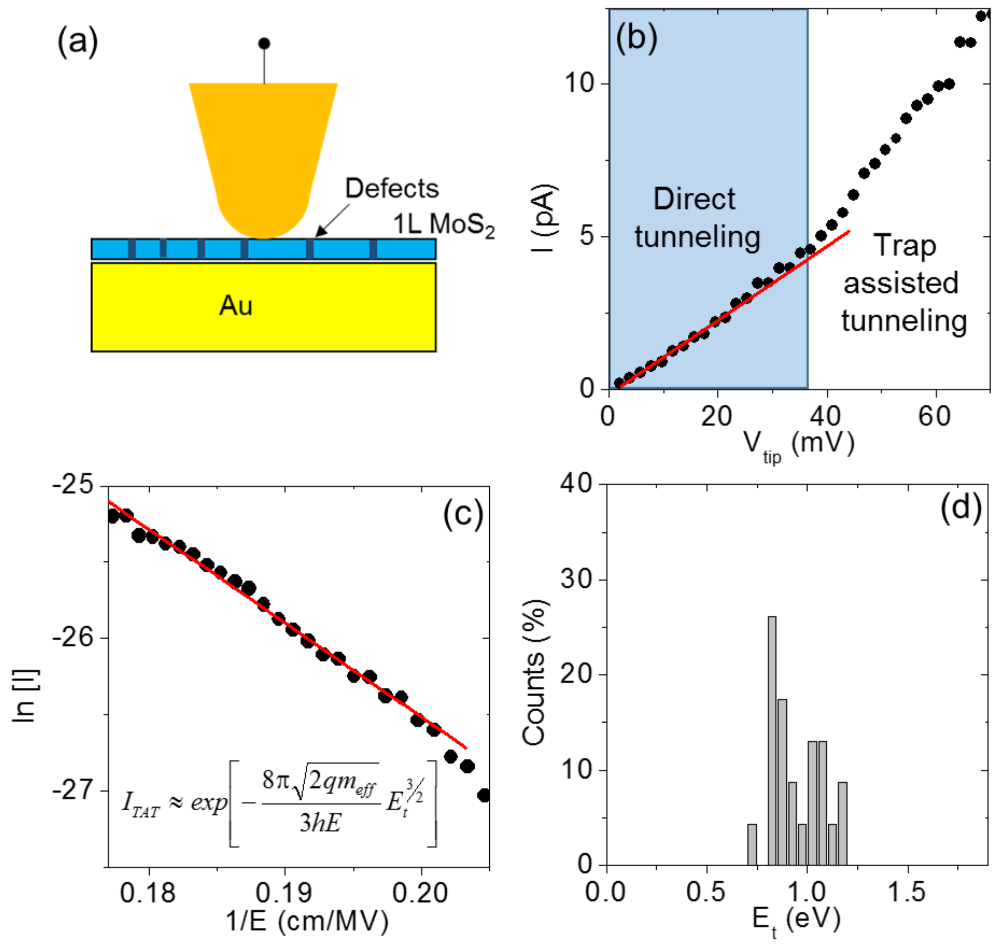

Figure S6 (a) Schematic of the tip/MoS_2/Au system, where the presence of defects (e.g. sulfur vacancies) in the 1L MoS_2 has been indicated. (b) Typical I-V_{tip} curve, where two conduction regimes are indicated: a linear regime (at lower bias) ruled by direct tunnelling, and an exponential regime (at higher bias) ruled by trap-assisted-tunnelling. (c) Plof of ln(I) vs 1/E, demonstrating that current can be described by the trap-assisted-tunnelling equation (in the insert). Here, the electric field E was evaluated as $E=(V_{tip}+V_{bb})/d$, where V_{tip} is the tip bias, d=0.65 nm is the thickness of 1L MoS_2 and V_{bb}=0.3V is the average value of the upward band bending of MoS_2 due to p-type doping induced by Au. The trap energy level E_t (referred to the conduction band edge) was evaluated from the linear fit. (d) Histogram of the E_t values obtained by fitting of all the I-V_{tip} curves in Fig.5(d) of the main manuscript.

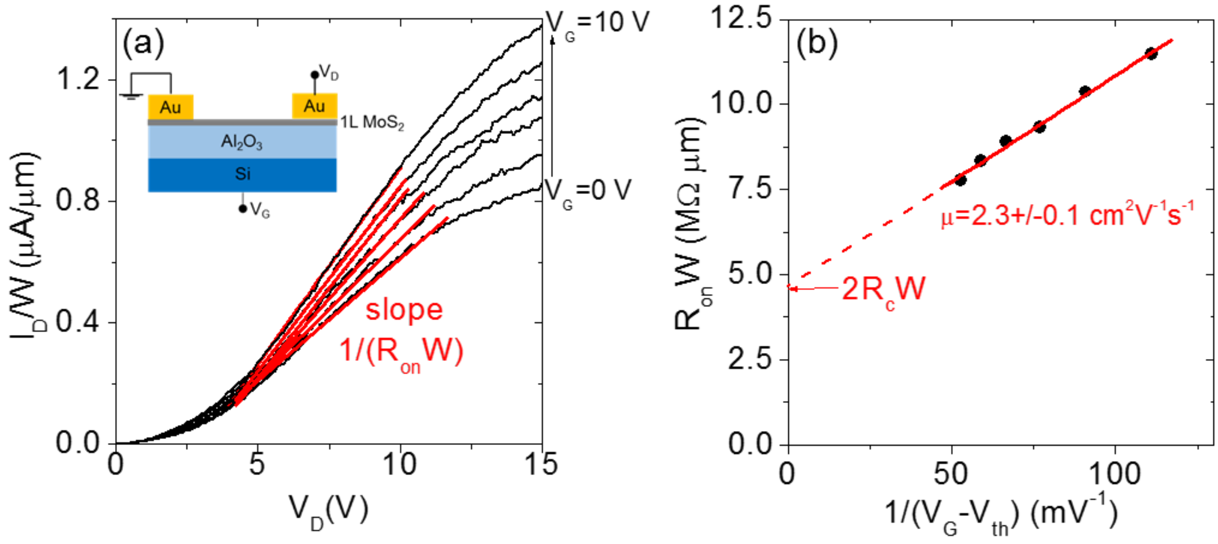

Figure S7 (a) Fitting of the linear region of the output characteristics (I_D/W vs V_D) of the back-gated 1L MoS_2 FET to evaluate the on-resistance R_{on}W. (b) Plof of R_{on}W vs 1/(V_G-V_{th}) and linear fit with the equation $R_{on}W=2R_CW+L/[\mu C_{ox}(V_G-V_{th})]$, where R_CW is the contact resistance, L=10μm is the channel length, μ is the electron mobility, C_{ox} is the capacitance density of 100 nm Al_2O_3, V_{th}=-9 V is the threshold voltage. A contact resistance R_CW≈2 MΩ μm and a mobility μ=2.3±0.1 cm^{2}V^{-1}s^{-1} were evaluated from the linear fit.
